# Good Practices in Sponge Natural Product Studies: Revising Vouchers with Isomalabaricane Triterpenes

**DOI:** 10.3390/md20030190

**Published:** 2022-03-04

**Authors:** Paco Cárdenas, Jayani Gamage, Chamari M. Hettiarachchi, Sunithi Gunasekera

**Affiliations:** 1 Pharmacognosy, Department of Pharmaceutical Biosciences, Uppsala University, Husargatan 3, 751 23 Uppsala, Sweden; jayani_gamage@ymail.com (J.G.); sunithi.gunasekera@farmbio.uu.se (S.G.); 2 Department of Chemistry, University of Colombo, Kumaratunga Munidasa Mawatha, Colombo 03, Sri Lanka; chamarih@chem.cmb.ac.lk

**Keywords:** Porifera, chemotaxonomy, isomalabaricane triterpenes, vouchers, misidentification, *Rhabdastrella globostellata*

## Abstract

Species misidentification in the field of natural products is an acknowledged problem. These errors are especially widespread in sponge studies, albeit rarely assessed and documented. As a case study, we aim to revisit reports of isomalabaricane triterpenes, isolated from four demosponge genera: *Jaspis*, *Geodia*, *Stelletta* and *Rhabdastrella*. From a total of 44 articles (1981–2022), 27 unique vouchers were listed, 21 of which were accessed and re-examined here: 11 (52.4%) of these were misidentified. Overall, 65.9% of the studies published an incorrect species name: previously identified *Jaspis* and *Stelletta* species were all in fact *Rhabdastrella globostellata*. We conclude that isomalabaricane triterpenes were isolated from only two *Rhabdastrella* species and possibly one *Geodia* species. In addition to shedding a new light on the distribution of isomalabaricane triterpenes, this study is an opportunity to highlight the crucial importance of vouchers in natural product studies. Doing so, we discuss the impact of species misidentification and poor accessibility of vouchers in the field of sponge natural products. We advocate for stricter voucher guidelines in natural product journals and propose a common protocol of good practice, in the hope of reducing misidentifications in sponge studies, ensure reproducibility of studies, and facilitate follow-up work on the original material.

## 1. Introduction

The necessity of publishing natural product (NP) studies with the right species name is obvious to any researcher, not just as a requirement of reproducibility, but also to make these studies trustworthy sources of data for other fields. However, previous articles have repeatedly felt the need to put forward the importance of taxonomy for NP studies [1] and expressed concerns about misidentification in the NP literature, whether it be plants [2,3] or sponges [4,5], one of the primary sources of new marine natural products [6]. Indeed, identification errors are acknowledged and especially widespread in sponge natural product studies, albeit rarely assessed and documented. Van Soest et al. (1996) [7] is the only study that tried to solve a sponge chemotaxonomy problem by re-examining several NP vouchers, essentially from studies from the 1980s–1990s. As expected, they found several misidentifications. Fortunately, because some of the authors were the original identifiers of several of these vouchers, they could easily access most vouchers; they do however mention that they could not access a few others. Indeed, now that most NP studies have made a habit of keeping a voucher and including a voucher number in their publications, one remaining issue is the poor accessibility and curation of vouchers (P.C., personal experience). Open and easy accessibility is an obvious requirement to serve the purpose of the voucher, even more so when considered in the context of the FAIR principles (Findable, Accessible, Interoperable, Reusable) for the management and sharing of data [8]. This study will assess voucher accessibility in sponge NP studies, using chemotaxonomy issues raised by isomalabaricane triterpenes (ITTs) as a test case.

**Figure 1:**
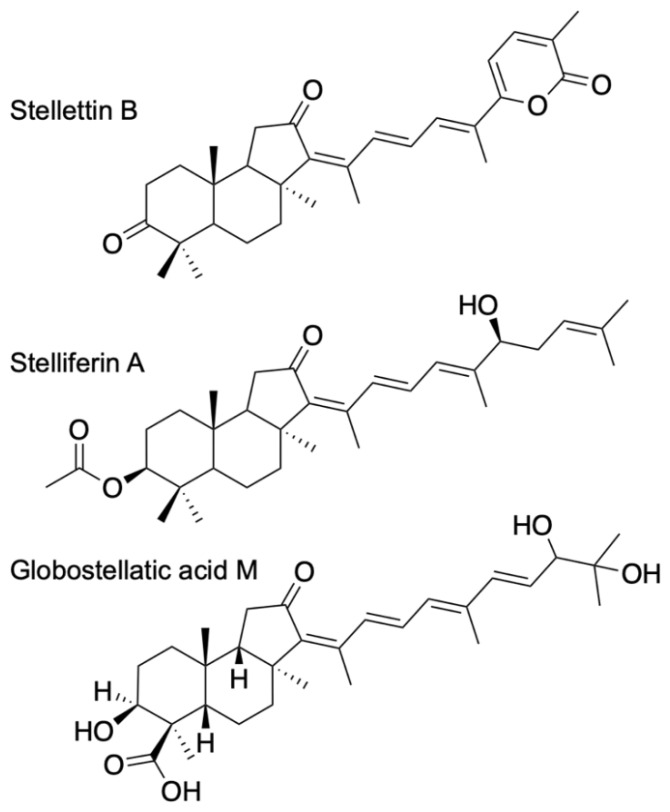
Examples of isomalabaricane triterpenes, often distributed in three groups: stellettins, stelliferins and globostellatic acids.

**Figure 2:**
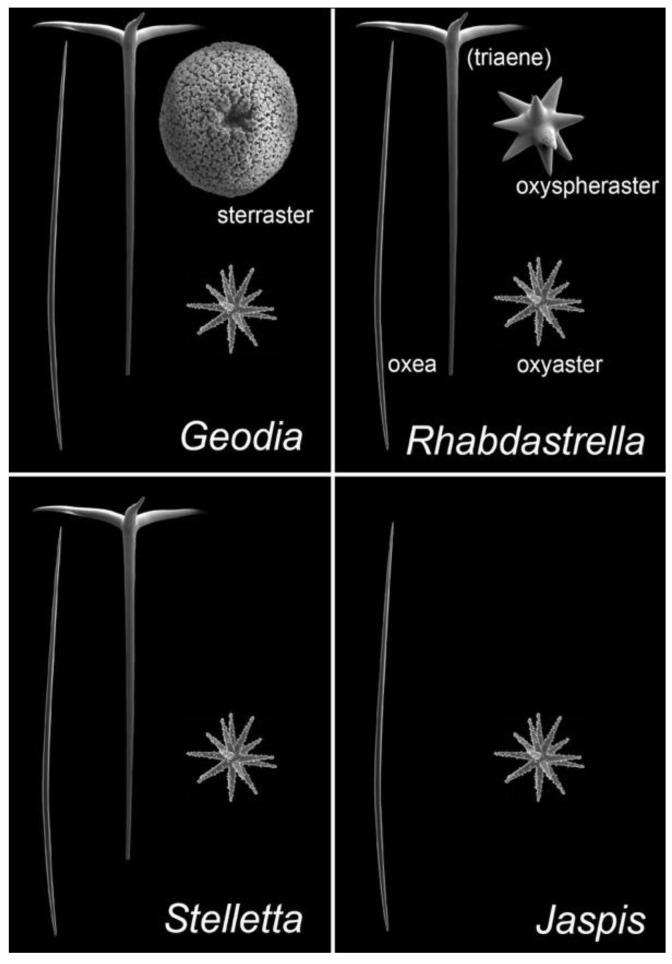
Comparison of siliceous spicule assemblages of demosponge genera in which isomalabaricane triterpenes have been reported: *Jaspis*, *Geodia*, *Stelletta* and *Rhabdastrella*. Names of the spicule types are given; ‘triaenes’ can be absent in some species and populations of *Rhabdastrella* so the term is in parentheses. Spicule pictures (taken with a scanning electron microscope) are representative of spicule types, they are here duplicated for pedagogical purposes; they are also not represented at the same scale.

ITTs are tricyclic terpenoids discovered in marine sponges by Ravi et al. (1981) [9]. Forty years later, dozens of studies have contributed to enrich this unique large family of sponge compounds, with more than 160 different structures, usually subdivided into three groups: stellettins (mainly with δ-lactone ring or carboxyl in side chain), stelliferins (oxygenated side chains) and globostellatic acids (carboxylation at C-4) (Figure 1), many of which have significant cytotoxic activities [10,11]. For example, stellettin B (Figure 1) is one of those promising compounds with high antitumor activities [12,13,14,15]. One of the richest species in terms of ITTs is undeniably the demosponge *Rhabdastrella globostelletta* (Carter, 1883) [16], present throughout the Indo-Pacific region [17] in shallow-water coral reef environments. Extracts of its yellow internal pigments led to the discovery of rhabdastrellic acid-A (=*E* isomer of stellettin G) and globostellatic acids [18,19]. So far, ITTs have been isolated from a handful of demosponge species belonging to four demosponge genera belonging to the Astrophorina suborder: *Jaspis*, *Geodia*, *Stelletta* and *Rhabdastrella* [11]. The odd distribution of ITTs in only a few species from four different species-rich genera with similar skeletons raises the possibility of misidentification. Indeed, demosponge species identification often relies on the categories and morphology of siliceous elements, called ‘spicules’, that make up the skeleton [20]. Considering the similar spicule assemblages of *Jaspis*, *Geodia*, *Stelletta* and *Rhabdastrella* (Figure 2), failure to observe one spicule category may easily lead to a wrong genus assignation (Figure 2), consequently leading to a wrong identification of the species. To make matters worse, secondary spicule losses through environmental conditions [21,22,23] or evolution [24] can easily mislead the identifier. Previous works have tried to solve small pieces of the taxonomic confusions in connection with *R. globostellata* and ITTs [25,26] but a complete and updated revision of the NP literature is required to verify the origin of these compounds. The aims of this study were to (i) revise the taxonomy of voucher specimens used in previous sponge NP studies on ITTs; and therefore (ii) revise the ITT diversity of these species, while (iii) assessing misidentification and voucher accessibility of these studies. Overall, this study is an opportunity to show in practice the crucial importance of vouchers in NP studies, and their accessibility status long after these studies are published. This is followed by a discussion on practices of sponge voucher handling and deposition, and possible ways to improve accessibility of these vouchers.

## 2. Results

### 2.1. Revision of Vouchers

**Figure 3:**
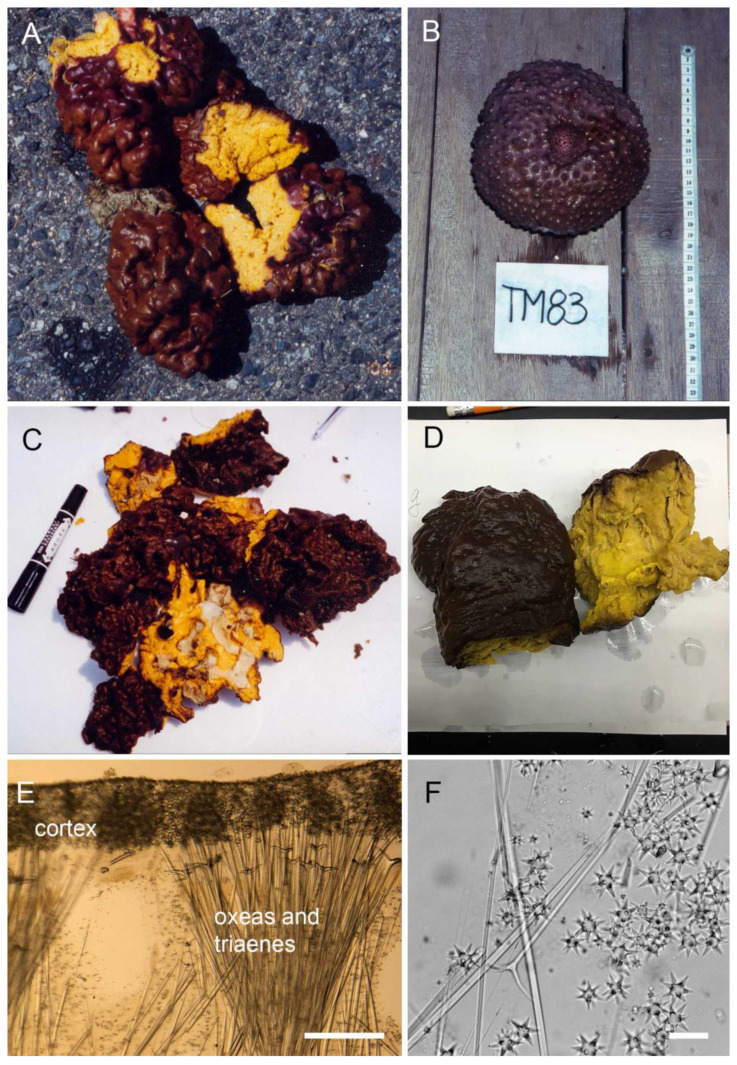
Pictures of specimens used in natural product studies on isomalabaricane triterpenes. (**A**) *Rhabdastrella globostellata* (original ID in article), Amami-oshima, Japan, voucher ZMAPOR 16401, Hirashima et al. [55] (Picture: T. Iwagawa). (**B**) *R. globostellata* (original ID in article), South Sulawesi, Indonesia, voucher ZMAPOR 17166, Fouad et al. [33] (Picture: R. A. Edrada). (**C**) *Rhabdastrella* aff. *distincta* (original ID in article) = *R. globostellata* (revised ID from this study), Hainan Island, China, voucher ZMAPOR 2717, Lv et al. [47] (Picture: J. Hiort). (**D**) *Rhabastrella* sp. before extraction, Lai et al. [27] (Picture: B.-R. Peng). (**E**) *R.* cf. *globostellata*, thick section of cortex showing a dense layer of star-shaped spicules at the surface (oxyspherasters) supported by larger spicules (oxeas and triaenes), voucher PDZ_1_98-1-10, Mindanao, Philippines, Tasdemir et al. [25]; scale: 400 µm. (**F**) Spicule preparation of *Stelletta tenuis* (original ID in article) = *R. globostellata* (revised ID from this study), Hainan Island, China, voucher HN1120-5, Li et al. [61]. Note the characteristic star-shaped spicules (oxyspherasters) of the genus *Rhabdastrella* and one large triaene; scale: 70 µm.

Table S1 lists relevant details of all 44 NP studies (1981–2022) on sponge ITTs as of January 2022, including 43 articles and one PhD thesis. Table S1 shows the compounds identified, the taxonomist who identified the material (when specified), the locality of the voucher, descriptive details about the voucher (from the article), the voucher number and where it is deposited, along with our revised identifications based on our examination and comparative material, and occasionally based on other taxonomists and/or the literature. Pictures of vouchers (external morphology and/or spicules) were added. For an easier overview and discussion of the results, Table 1 summarizes the information from Table S1. Of the 44 studies, seven do not have any vouchers. Lai et al. [27] report a voucher #2017-1221-SP but after examination we discovered that this voucher was actually not from the specimen studied. Indeed, a neighboring specimen, from a different species, was mistakenly photographed and collected as a voucher (K-H Lai, pers. comm.) so there is also no voucher for this study. Within the remaining 36 studies with vouchers, eight vouchers were used for two to three consecutive articles (Table S1), so in the end 26 unique vouchers were recorded. A total of 21 vouchers were re-examined for this study, some illustrated in Figure 3: 16 vouchers were received at Uppsala University (Sweden). We were sent pictures of specimens and/or spicule preparations for another five vouchers (Table S1). Five vouchers, all from Hainan Island, China, could not be re-examined: *Geodia japonica* [28], *Stelletta tenuis* [29,30] and *Jaspis stellifera* [31,32]. It is believed the voucher of *G. japonica* is lost: it was kept at the ‘Research Center of Organic Natural Products Chemistry, Sun Yat-Sen University, Guangzhou, China’ which is now closed (Prof Jun Xu, pers. comm.). The *S. tenuis* voucher from Su et al. [29] was stored at the ‘Research Center of Organic Natural Products, Zhongshan University, Guangzhou, China’, but we were unable to locate this center. As for the three other vouchers, the authors could not locate the material or could not find information/contacts about the institutes holding the vouchers. Of the 16 vouchers received, six were spicule preparations (all from the Naturalis Museum, Leiden, The Netherlands), nine were subsamples in EtOH or dried after EtOH extraction and one was from grinded powder (from the Muséum National d’Histoire Naturelle, Paris, France). Importantly, three NP studies out of 44 described the voucher in sufficient detail (external morphology and spicule measurements) so that we could confidently confirm [33,34] or revise [35] the identification before even seeing the voucher.

The spicules of all vouchers received were examined and measured, and compared with holotypes (*Rhabdastrella globostellata*, *Rhabdastrella providentiae*, *Rhabdastrella distincta*, *Stelletta tenuis*), comparative material and taxonomy literature. Out of the 21 vouchers re-examined, 11 (52.4%) were misidentified. Specimens identified as *Jaspis* sp., *Jaspis stellifera*, *Stelletta* sp., *Stelletta tenuis*, *Geodia globostellifera* were all *R. globostellata*. On the other hand, *R. globostellata* ZMAPOR 15784a from Sulawesi (Indonesia) was re-identified as *Rhabdastrella* sp.; it is not *R. globostellata* but a currently undescribed species. The remaining nine vouchers were all confirmed to be *R. globostellata*. *Rhabdastrella* sp. from Taiwan (Figure 3D) is probably *R. globostellata*, based on its external morphology, but we cannot be entirely sure since there is no voucher. Since *S. tenuis* HN1120-5 [61] was re-identified as *R. globostellata* (Figure 3F) and since all *S. tenuis* identifications stem from the same taxonomist (Table S1), and were published with similar co-authors [29,30,61], one can reasonably consider the other *S. tenuis* records to be misidentifications of *R. globostellata*. Seemingly, most *J. stellifera* and *Jaspis* sp. identifications stem from the same authors [31,32,49] who misidentified *Jaspis* sp. voucher HSC-39 [49,50,51], which we re-identified as *R. globostellata*. Therefore, we can confidently conclude that all these *Jaspis* specimens are *R. globostellata* as well. Further evidence comes from the distribution of these species. So far, *J. stellifera* has been formally reported only from South Australia and Tasmania [26] suggesting that it is a temperate species. Therefore, the distributions of *R. globostellata*, a tropical/subtropical species, and *J. stellifera* do not overlap, which further suggests that all records of *J. stellifera* in the South China Sea are misidentifications. Following similar reasoning, we consider that NP studies without vouchers using the name *Jaspis* sp. or *J. stellifera*, from localities where it has never been observed but where *R. globostellata* is common, are all misidentifications of *R. globostellata* [9,36,38]. Despite not being able to revise the *G. japonica* voucher (presumably lost) from shallow waters of Hainan Island, we are confident that this is a misidentification as well since *G. japonica* is a temperate deep-sea species only formally recorded from Japan, Korea and Alaska [67,68,69,70]. We assume the original material could still have been a *Geodia* since this genus has very characteristic ball-shaped spicules (sterrasters in Figure 2) that would be difficult to misinterpret for any sponge taxonomist. We are left with the *Stelletta* sp. from Somalia [37] with no voucher. However, the Somalian coast is in the distribution range of *R. globostellata,* and the authors note the external (brown) and internal (yellow) colors which match with those of *R. globostellata* (Figure 3). It is, therefore, reasonable to think that this is also a misidentification, although we cannot be fully sure without a voucher. Overall, 29 (65.9%) articles out of the 44 publications were published with a wrong species name. According to our revised identifications, ITTs were isolated only from two species of *Rhabdastrella* (*R. globostellata* and *Rhabdastrella* sp. Indonesia) and one putative species of *Geodia* (*Geodia* sp. from the South China Sea). All ITT reports from *Stelletta* or *Jaspis* were misidentifications. Following this revision, Table 2 compiles the current chemical diversity of species with ITTs.

### 2.2. NP Journal Taxonomy/Voucher Guidelines

We reviewed the taxonomy/voucher guidelines for 11 NP journals that currently publish most sponge articles. Seven of these have no guidelines or requirements for vouchers or taxonomic identifications: *Tetrahedron Letters, Tetrahedron, Bioorganic & Medicinal Chemistry Letters, Natural Product Communications, Bioorganic & Medicinal Chemistry, Natural Product Research* and *Steroids*. Only four journals mention voucher guidelines: *Marine Drugs, Journal of Natural Products, Phytochemistry Letters* and *Molecules*. Guidelines in *Marine Drugs* and *Molecules* (both MDPI publications) are identical and only refer to “*research involving plants*”, with no mention of marine organisms. Although MDPI guidelines request GPS coordinates of the locality and that “*voucher specimens must be deposited in an accessible herbarium or museum*”, some sponge NP articles from these journals do not follow these guidelines [75,76,77]. *Phytochemistry Letters* has clear guidelines in terms of deposition of the plant vouchers in an herbarium, checking the validity of the species name and including an illustration if the species is poorly known. Although they do not clearly transfer these requirements to marine organisms, authors must consider that it applies to sponges as well. However, recent sponge NP studies in *Phytochemistry Letters* have not had their specimens identified by a sponge taxonomist and more importantly have not deposited their vouchers in a museum [62,78,79]. *Journal of Natural Products* has detailed guidelines to report the species identification; if the specimen is not identified to the species level, a description is required; the identifier should be an expert in the organisms studied, but they do not require the publication of the name of the identifier; the voucher should be deposited in a “*herbarium*” (but do not mention museums for animal material); the GPS coordinates are only recommended. Some sponge studies in *Journal of Natural Products* are still depositing vouchers in university or lab collections [80,81].

## 3. Discussion

### 3.1. *Rhabdastrella globostellata* Is a Variable Species

By collecting vouchers and voucher pictures, we noticed some shape variations in *R. globostellata*. This species is usually a massive subglobular species which can be cup-shaped in large specimens (Figure 3B), but a morphotype with a convoluted surface (brain-like) seemed also quite common (Figure 3A) in the South and East China Sea: ZMAPOR 16401 from Hirashima et al. [55], ZMAPOR 12451 from Rao et al. [19], PIBOC O38-301 from Kolesnikova et al. [35,65], HM06-2016.1 from Kiem et al. [62] and NCCT-B139 from Trang et al. [66]. Morphology and measurements of the spicules from both morphotypes did not reveal any consistent differences. We wonder whether the brain-like morphotype could represent a separate cryptic lineage, but molecular markers are required to further test this. 

Spicule variations of *R. globostellata* were observed for other specimens. We confirm that triaene spicules (Figure 1) were overall vestigial or absent in vouchers from Western Pacific subtropical populations, a variation mentioned already [26]. Since *Jaspis* is a genus without triaenes (Figure 1), this loss of triaenes is the main reason *R. globostelletta* has often been mistaken for a *Jaspis* in these regions. This is how *R. globostelletta* started to be wrongly identified as *J. stellifera* in the Great Barrier Reef [82]. The loss of triaenes is actually a common feature of several other *Rhabdastrella* species (e.g., *R. cribriporosa, R. distincta*).

### 3.2. ITTs Are Chemotaxonomy Markers

Our results also shed light on the chemotaxonomy of *Rhabdastrella*. Van Soest & Braekman [4] had suggested ITTs were markers of the Ancorinidae family in which we find *Stelletta, Jaspis* and *Rhabdastrella*. Later, acknowledging some misidentifications, Tasdemir et al. [25] claimed that “isomalabaricanes are chemotaxonomic markers for *R. globostellata*”. However, our results invalidate this claim and confirms the presence of ITTs in at least two *Rhabdastrella* species—*R. globostellata* and *Rhabdastrella* sp. Indonesia—as well as one *Geodia* sp. from the South China Sea. A shared chemical marker between *Rhabdastrella* and *Geodia* is not unexpected as they have shown to be phylogenetically close [24]. More *Geodia* species will need to be tested for the presence of ITTs to confirm this association.

Table 2 compiles the resulting chemical diversity of species with ITTs. *R. globostellata* is by far the richest species in terms of ITTs diversity with ~160 different ITTs in this single species. Future work will now need to investigate if different chemical lineages could be revealed in the Indo-Pacific region, which could eventually be correlated with cryptic genetic lineages. The true producer of these ITTs, sponge and/or microbial symbiont is presently unknown. The microbiome of *R. globostellata*, a High Microbial Abundance (HMA) sponge, was studied with 16S amplicon sequencing on specimens from Guam [83,84] and Vietnam [85]. In the specimens from Guam, 23 microbial phyla were detected in the microbiome, the most abundant ones being Acidobacteria, Chloroflexi SAR202, Cyanobacteria, Gemmatimonadetes and Alphaproteobacteria; the predominant bacterium was a Cyanobacteria highly similar to *Candidatus* Synechococcus spongiarum [84]. Cultured bacterial symbionts have been shown to produce small molecules, including diketopiperazines (Table 2) [73,74], but no ITTs so far. The sharing of ITTs between *R. globostellata*, *Rhabdastrella* sp. Indonesia and *Geodia* sp. therefore suggests either phylogenetic closeness or the sharing of a highly specific symbiont. Our results also have repercussions for other NP articles reporting sterol and fatty acid composition in *J. stellifera* [71,72,86]. For the same reasons given in the present study and previous ones [26,87], we are confident that these studies are also on misidentified *R. globostellata*.

### 3.3. Sponge Misidentifications Have Multiple Reasons 

Sponges are admittedly a challenging group to work with in taxonomy. Thanks to an increasing amount of molecular data, the traditional classifications have seen some drastic changes these last years, in the four classes of sponges: Demospongiae [88,89], Hexactinellida [90], Calcarea [91,92] and Homoscleromorpha [93]. These changes, added to the subtleties and caveats of spicule terminology, the dense taxonomy literature (not free of identification errors) make sponge taxonomy a challenging field for a non-specialist. However, reproducibility and reusability of NP results do rely on rigorous taxonomic identification of the specimens investigated, so that for example promising bioactive compounds can be re-isolated from the same organisms. Our results show that a very high number of articles (29, 65.9%) were published with a wrong genus/species name (Table 1). However, to identify the specimens, most of these papers (22) collaborated with a demosponge taxonomist (i.e., a taxonomist with a track record of publications in that field) while 14 articles collaborated with a more general taxonomist (Table 1). Demosponge taxonomists were mistaken 11 times (out of 22), which in itself highlights the challenge of sponge identification, even for experts. None of the general taxonomists got the species name right. Reasons why experts make mistakes is species morphological variability (cf. Section 3.1) and poor description of species from the 19th century where the type material has never been revised, which was the case for *Jaspis stellifera* and *Stelletta tenuis*. Cascading errors in sponge NP publications are often the result of an original error from an identifier, repeated along a series of articles usually from the same lab or authors (Table S1). For instance, from the 1970s to the 1990s, all the *Rhabdastrella globostellata* that were collected at Heron Island, Queensland (Eastern Australia) were misidentified as *J. stellifera* following the demosponge taxonomist Patricia R. Bergquist [82]. This explains why Australian taxonomists continued using *J. stellifera* or *Jaspis* sp. for *R. globostellata* from Japan. Kennedy [26] revealed the mistake, relayed by Tasdemir et al. [25] to the NP community but the name *J. stellifera* was inappropriately used again more recently on material from the South China Sea [31,32,59,60]. Another misidentification name for *R. globostellata* is *S. tenuis* [29,30,61], also appearing in sponge microbiology publications [73,94,95]. We have examined the *S. tenuis* voucher HN1120-5 [61] (Figure 3F). Based on its spicules and comparison with the holotypes of *S. tenuis* and *R. globostellata*, it is without any doubt *R. globostellata*. Actually, *S. tenuis* has never been formally reported since its original description in Java in 1897 [96], therefore all publications mentioning *S. tenuis* without a clear description of the specimen are suspicious and most likely all refer to *R. globostellata*.

### 3.4. The Accessibility of Vouchers Is Crucial 

The fact that even sponge taxonomists misidentified some specimens (Table 1) highlights the crucial importance of depositing a voucher for every published study, so that identifications can be revised. Most NP studies after the 1990s do have a voucher with a collection number. However, a voucher is not sufficient, as we found that access to vouchers is also crucial. Indeed, as a byproduct of the study, one should consider vouchers as data in itself, which should therefore ideally follow the FAIR principles (Findable, Accessible, Interoperable, Reusable) [8]. The accessibility of vouchers deposited at universities, institutions or lab collections can be increasingly difficult with time. Vouchers stored in labs, university departments or even institutes do not ensure that they will be preserved and easily accessible to the scientific community after some years. Many of these collections risk being lost with time, movement/retirement of researchers or restructuration of labs. In the case of ITT sponge vouchers, many voucher fragments were fortunately sent for identification to taxonomists R. van Soest and N. de Voogd (Naturalis Museum, The Netherlands), so that we were able to access them easily through the museum collections. Sponge vouchers should ideally be stored in national or city museums, where long-term curation and accessibility to international scientists will be guaranteed, and will be an easy process. Only 14 (53.8%) out of the 26 vouchers were stored in a museum (Table 1). The other 12 vouchers coming from 2002–2022 publications were stored in university or lab collections and were obtained by contacting the authors. We were able to access all vouchers in museum collections, but only eight of the 12 vouchers stored in university/institute collections (Table 1) and that is sometimes only possible with substantial detective work to locate the vouchers or the authors. We envision that with time, these 12 vouchers will become less accessible, as the authors move on and as labs are restructured or dismantled. Of course, this does not concern large well-curated NP collections/platforms which ensure from the start proper voucher handling/curation and potential long-term storage (e.g., NatureBank, https://www.griffith.edu.au/institute-drug-discovery/unique-resources/naturebank (accessed 1 March 2022); the Harbor Branch Oceanographic Institute Marine Biotechnology Reference Collection (HBOI MBRC) http://hboi-marine-biomedical-and-biotechnology-reference-collection.fau.edu/app/data-portal (accessed 1 March 2022); the National Cancer Institute (NCI) Natural Products Repository https://dtp.cancer.gov/organization/npb/introduction.htm (accessed 1 March 2022)). However, to our knowledge, none of these large collections take in vouchers from other studies. 

By losing a voucher, one loses proof of the source organism, which could make the study non-reproducible and the compound not findable again. For example, since the *Geodia* from Zhang & Che [28] was most probably misidentified, the loss of the voucher means we do not know which *Geodia* species produces ITTs and if it is a *Geodia* indeed. Apart from hampering reproducibility, misleading future NP or chemotaxonomy studies, and just generally minimizing the scientific impact of the study, one should not forget as well the many repercussions of these misidentifications. For example, they partly invalidate the many chemistry review articles, e.g., on compounds from *Jaspis* [97] or *Stelletta* [98] which repeatedly propagate the same errors. This may for example lead to false scientific questions such as trying to reconcile the shared appearance of ITTs in *Jaspis, Stelletta* and *Rhabdastrella* species. Overall, these errors will become a source of recurrent confusion until corrected and revised. Additionally, with scientific advancements and access to new technologies, techniques such as genome mining to unravel the biosynthetic pathways of natural products are becoming common. Misidentifications will waste money, time and hamper such possibilities because scientific efforts could be wasted on the wrong species. In a time when ‘drug repurposing’ is a hot area for natural products, knowing which organism actually produces the compound is vital to establish feasible methods of production. The problem of sponge misidentification has been raised previously in the field of NPs [4,5]. The present study is, to our knowledge, only the second [7] to revise a set of sponge NP vouchers to solve putative inconsistencies, and therefore assess the problem of misidentification in the field of sponge NPs. This study is however the first to highlight the problem of voucher accessibility in the field of sponge NPs.

### 3.5. Recommended Protocol for Sponge NP Studies

To reduce future widespread misidentification issues in the field of sponge NP studies, we would like to take the opportunity of this study to (i) recommend a common (ideal) protocol for reporting of sponges for NP studies and (ii) advocate for stricter guidelines for NP journals publishing these works, as well as for higher expectations from peer reviewers, thereby improving the general standards of the field. With more and more sponge NP studies integrating sponge genomic data, it goes without saying that vouchers are also to be kept and deposited for sequenced specimens. We are aware of the difficulties of fieldwork which sometimes limit what can be done but sponge and sponge-associated microbe NP studies should try to follow as much as possible this protocol to maximize the scientific impact and reproducibility of their study.

1. Upon collection, take pictures of the whole specimen (external and internal appearance). A picture of the specimen is priceless for a sponge taxonomist and can already give a lot of information about the species identity (shape, external/internal color). Usually, the voucher is a small subsample so one loses substantial information about the external morphology if a taxonomist only examines the voucher.
2. Prepare the voucher from the start, upon collection of the specimen in the field. Just after collection, cut a small piece (1 cm × 1 cm) including the surface of the sponge. Place the piece in 10 times its volume of EtOH 96% to preserve the DNA and the morphology. Change the EtOH at least twice, after ~1 h and after ~6 h. Of course, a larger voucher is perfectly ok, but then increase the amount of EtOH 96% accordingly to properly preserve the DNA (that being said, DNA is usually preserved better in a small piece). With this mode of preservation, the voucher could be used in the future for morphology and molecular work (e.g., barcoding, population genetics, genomics, etc.) and it is easily stored at room temperature.
3. A voucher should have the following metadata recorded and reported in the publication: date of collection, collector, locality name, GPS coordinates, depth and habitat (e.g., coral reef, seagrass bed, mangrove, rocky bottom) of the locality where the specimen was collected. These descriptors will be useful for several metadata analyses (e.g., [99]) as well as for the taxonomist.
4. Collaborate with a sponge taxonomist for the identification. Indicate in the publication the name of the taxonomist identifier. Sponge experts can be found on the World Porifera Database (WPD, http://www.marinespecies.org/porifera, accessed 1 March 2022): WPD editors are listed on the home webpage, and additional experts are listed in the “Who is who in sponge science” list [100].
5. Check the validity and spelling of the species name on the WPD before publishing. All species and their taxonomy are recorded and updated in the WPD, part of the World Register of Marine Species (WoRMS).
6. With the help of the sponge taxonomist, include in the publication a short description of specimen with (i) external and internal color (the best is a picture), (ii) overall shape, and if possible, (iii) spicule repertoire with spicule measurements. It will significantly help other taxonomists to trust or not the identification. These descriptions can be in the Materials section (e.g., [33,101]) or in the Supplementary Information (e.g., [35]). This description should be mandatory if the specimen is identified to the genus level only. Additionally, including a molecular barcode of the specimen in the publication (and deposited in Genbank) can be very useful: 28S and COI (cytochrome c oxidase 1) are the two most widely used barcodes to help with sponge identification.
7. Deposit the sponge voucher in a recognized national or city museum with a unique museum number to be included in the publication. The sponge taxonomist collaborator may help you with this last step.

To conclude, species misidentification may always exist in sponge NP studies, but one of the best solutions to promote reproducibility and corrections of errors is for NP journals to require for publication no less than (i) a description of the sponge (picture included) and (ii) that vouchers be deposited in a recognized museum.

## 4. Materials and Methods

### 4.1. Abbreviations

NHM, Natural History Museum, London, UK; UPSTZY, type collection of the Zoological Museum of Uppsala, Uppsala, Sweden; ZMAPOR, Amsterdam Porifera collection, now stored at Naturalis, Leiden, The Netherlands; ZMB, Museum für Naturkunde, Berlin, Germany.

### 4.2. Comparative Material and Vouchers

After compiling all NP articles reporting ITTs (until January 2022), a list was obtained of eight sponge species from four genera (*Rhabdastrella globostellata* (Carter, 1883), *Rhabdastrella providentiae* (Dendy, 1916), *Rhabdastrella* aff. *distincta* (Thiele, 1900), *Geodia japonica* (Sollas, 1888), *Geodia globostellifera* Carter, 1880, *Stelletta tenuis* Lindgren, 1897, *Stelletta globostellata* Carter, 1883, *Jaspis stellifera* (Carter, 1879)) and three undetermined species (*Stelletta* sp., *Jaspis* sp., *Rhabdastrella* sp.). Undetermined species were used in different articles so they could actually represent different species. We checked the validity of each of the eight species names in the World Porifera Database (WPD, http://www.marinespecies.org/porifera, accessed 1 March 2022), part of the World Register of Marine Species (WoRMS). Species were all valid except for *S. globostellata*, which is a junior synonym of *R. globostellata* (i.e., the species is correct, but the genus is not correct anymore). To revise the identification of vouchers, comparative material was necessary and ideally one would try to compare with the species holotypes, which are the specimens from the original descriptions. The following holotypes were therefore obtained on loan from museums: *Rhabdastrella globostellata* (NHM 1883.5.3.1, Galle, Sri Lanka, slide), *Rhabdastrella providentiae* (NHM 1920.12.9.129 and 1920.12.9.74a, Providence Island, Seychelles, slides), *Rhabdastrella distincta* (ZMB 3190, Ternate, Indonesia, slides) and *Stelletta tenuis* (UPSTZY 2099, Java, Indonesia, wet specimen and slides). We also relied on the re-descriptions of the holotypes of *R. globostellata* and *J. stellifera* [26]. Re-identification of vouchers were done by PC and JG.

Some studies did not have a voucher (essentially articles from the 1980s and 1990s). For the other studies, sponge vouchers and/or pictures of vouchers were obtained by emailing authors or institutes/museums where vouchers were deposited, as described in the articles. We have also in some cases contacted the taxonomist who identified the material (when cited in the article) since they would often keep a spicule preparation or notes of what they identify. It is worthy of note that some NP publications included a more or less short description of the specimen so that it was directly possible to confirm or refute some of the identifications, before examining the voucher.

### 4.3. Spicule Preparations 

Spicule preparations of the vouchers were prepared by digesting a piece of sponge (5 × 2 mm) in chlorine in a 1.5–2 mL Eppendorf tube. One voucher received as grinded powder was treated the same way by placing a few mg of powder in chlorine. When the tissue was entirely dissolved, the remaining spicules were washed successively with H_2_O, 50% EtOH and 100% EtOH. A drop of the spicule solution on a glass slide was left to dry, on a hot plate. Then the spicules were covered with Eukitt^TM^ mounting medium (Sigma-Aldrich, St Louis, MO, USA) and a cover slide to make permanent slides. Spicules were examined with a light microscope, measured using an eye-piece micrometer, and documented by taking pictures with a camera connected to the microscope.

### 4.4. Voucher Guidelines in NP Journals

To assess sponge voucher recommendations in NP journals, we focused on journals that currently publish most sponge NP studies. For this, we searched for NP journals that published >10 articles with “marine sponge” in their title between January 2010 and April 2021. We retrieved 11 journals, here sorted according to the number of sponge NP articles found: *Marine Drugs* (175), *Journal of Natural Products* (117), *Tetrahedron Letters* (66), *Tetrahedron* (65), *Bioorganic & Medicinal Chemistry Letters* (64), *Natural Product Communications* (41), *Bioorganic & Medicinal Chemistry* (39), *Natural Product Research* (32), *Phytochemistry Letters* (19), *Molecules* (15) and *Steroids* (12). Author guidelines in these 11 journals was reviewed, with respect to taxonomy and vouchers.

## Figures and Tables

**Table 1:** Revision of *Rhabdastrella/Geodia/Stelletta/Jaspis* vouchers from natural product articles on isomalabaricane triterpenes. Studies using the same voucher are grouped together. Y/Yes; N/No; -/not applicable. For more details, see Table S1.

Ref.	Identifier	Description of Voucher 1. Picture 2. Color 3. Spicules 4. Barcodes	Voucher (Y/N)	Voucher in Museum (Y/N/-)	Voucher Accessed (Y/N/-)	Source Original ID	Source Revised ID	Misidentification (Y/N/?)
Ravi et al. (1981) [9]	-	2	N	-	-	*J. stellifera*	*R. globostellata*	Y
Ravi & Wells (1982) [36]	-	-	N	-	-	*J. stellifera*	*R. globostellata*	Y
McCabe et al. (1982) [37]	Bergquist	2	N	-	-	*Stelletta* sp.	*Rhabdastrella* ?	Y
Tsuda et al. (1991) [38]	Fromont	2	N	-	-	*J. stellifera*	*R. globostellata*	Y
Su et al. (1994) [29]	JH. Li	-	Y	N	N	*S. tenuis*	*R. globostellata*	Y
Ryu et al. (1996) [18], Oku et al. (2000) [39]	Van Soest	-	Y	Y	Y	*S. globostellata*	*R. globostellata*	N
Kobayashi et al. (1996) [40]	Fromont	2	N	-	-	*J. stellifera*	*R. globostellata*	Y
McCormick et al. (1996) [41], McKee et al. (1997) [42]	Pomponi	2	Y	Y	Y ^2^	*Stelletta* sp.	*R. globostellata*	Y
Rao et al. (1997) [19]	Van Soest	2	Y	Y	Y ^1^	*R. globostellata*	-	N
Bourguet-Kondracki et al. (2000) [43]	Lévi	-	Y	Y	Y	*R. globostellata*	-	N
Zampella et al. (2000) [44]	Hooper	-	Y	Y	Y ^2^	*Jaspis* sp.	*R. globostellata*	Y
Tabudravu & Jaspars (2001) [45]	Hooper	-	Y	Y	Y	*G. globostellifera*	*R. globostellata*	Y
Zhang & Che (2001) [28]	Chupu	-	Y	N	N	*G. japonica*	*Geodia* sp.?	Y
Meragelman et al. (2001) [46]	Kelly	-	Y	Y	Y ^2^	*Jaspis* sp.	*R. globostellata*	Y
Tasdemir et al. (2002) [25]	Harper	-	Y	N	Y	*R. globostellata*	*Rhabdastrella* cf. *globostellata*	N
Lv et al. (2004) [47], Lv et al. (2008) [48]	Van Soest	-	Y	Y	Y ^1^	*R.* aff. *distincta*	*R. globostellata*	Y
Tang et al. (2005) [49] Tang et al. (2006) [50], Tang et al. (2007) [51]	Van Soest? ^3^	-	Y	N	Y	*Jaspis* sp.	*R. globostellata*	Y
Clement et al. (2006) [52]	Kelly	1	Y	Y ^1^	Y ^2^	*R. globostellata*	-	N
Fouad et al. (2006) [33]	Van Soest	3	Y	Y	Y	*R. globostellata*	-	N
Agrawal (2007) [53]	-	-	N	-	-	*R. globostellata*	-	-
Aoki et al. (2007) [54]	de Voogd	-	Y	Y	Y	*R. globostellata*	*Rhabdastrella* sp.	Y
Lin et al. (2007) [30]	JH. Li	-	Y	N	N	*S. tenuis*	*R. globostellata*	Y
Hirashima et al. (2010) [55]	Van Soest	-	Y	Y	Y	*R. globostellata*	-	N
Li et al. (2010) [56] Li et al. (2012) [57]	de Voogd	1	Y	Y	Y	*R. globostellata*	-	N
Tanaka et al. (2011) [34]	Fromont	2, 3	Y	Y	Y	*R.* cf. *globostellata*	*R. globostellata*	N
Tang et al. (2012) [31]	-	-	Y	N	N	*J. stellifera*	*Rhabdastrella* sp.	Y
Xue et al. (2013) [58]	JH. Li	-	Y	N	Y ^2^	*Stelletta* sp.	*R.* ?*globostellata*	Y
Jin et al. (2014) [32], Xu et al. (2016) [59], Xu et al. (2018) [60]	Tang	-	Y	N	N	*J. stellifera*	*Rhabdastrella* sp.	Y
Li et al. (2015) [61]	JH. Li	-	Y	N	Y	*S. tenuis*	*R. globostellata*	Y
Kiem et al. (2018) [62], Dung et al. (2018a) [63], Dung et al. (2018b) [64]	Thun	1	Y	N	Y	*R. providentiae*	*R. globostellata*	Y
Kolesnikova et al. (2019) [35] Kolesnikova et al. (2021) [65]	Grebnev	1, 2, 3	Y	N	Y	*Stelletta* sp.	*R. globostellata*	Y
Lai et al. (2021) [27]	HH. Li	1 ^4^	Y ^4^	-	-	*Rhabdastrella* sp.	-	N
Trang et al. (2022) [66]	-	4	Y	N	Y	*R. globostellata*	-	N

^1^ Museum voucher not specified in the paper. ^2^ Specimen accessed second-hand through the notes, pictures and/or observations of another taxonomist. ^3^ Although R. van Soest is cited as the identifier, there is no voucher and no record of this specimen in the sponge collections in Amsterdam, so this information is considered doubtful (R. van Soest, pers. comm.). ^4^ Voucher #2017-1221-SP is mentioned by Lai et al. [27], but after examination we concluded this voucher was a very different sponge than the one studied. A mistake during collection of the voucher (K-H Lai, pers. comm.) leads to no voucher for this study. The 18S sequence of the original material was unfortunately lost (K-H Lai, pers. comm.). However, the original voucher was photographed before being extracted (Figure 3D): it looks like *R. globostellata,* but we cannot be sure since there is no voucher. We prefer to keep it identified as *Rhabdastrella* sp.

**Table 2:** Chemical diversity of *Rhabdastrella* and *Geodia* species with isomalabaricane triterpenes. Classification of isomalabaricane triterpenes follows [11]. For references to these compounds, see Table S1.

Species	Compounds
*Rhabdastrella globostellata*	**Isomalabaricane triterpene groups (ITTs)** **Stellettins**^1^ Stellettins A-R, W ^2^ (–)-Stelettin E Rhabdastrellic Acid-A (=*E* isomer of stellettin G) 22,23-dihydrostellettins B, D Rhabdastrellins A-F Jaspiferins C-H **Stelliferins** Stelliferins A-N 29-hydroxystelliferin A, D, E 3-*Epi*-29-hydroxystelliferin A, E 3-*Epi*-29-acetoxystelliferin E Stelliferin A, D, ribosides Geoditins A-B (23*E*) Isogeoditins A-B (23*Z*) 13-(*E*)-isogeoditin A **Globostellatic acids** Globostellatic acids A-M Globostelletin A-I 13,17-Globostellatic acid X methyl esters Globostellatic acid F methyl ester Globostellatic acid B methyl ester
	**ITT derivatives** Aurorals 1-4 (derivatives of globostellatic acids) Cyclobutastellettolides A and B Globostelletins A-I (derivatives of stellettin E?) Globostelletins J-R (cyclopentane side chain) Jaspiferals A-G (derivatives of globostellatic acids) 3-*O*-Acetyl-jaspiferals B/D/E methyl ester Jaspiferins A-B, I-J (derivatives of stelliferins?) Jaspiferoic acids A–B dimethyl esters (derivatives of globostellatic acids) Jaspolides A-B Jaspolides C-F (derivatives) Jaspolides G-H (dimeric) Rhabdastins A-G (derivatives of stelliferins?) Rhabdastrellins G-K Rhabdaprovidines A-C (biochemical degradation of ITTs?) Rhabdaprovidines D-G Stellettins S-V (nor-terpenoids) Rhabdaglostelones A-C (tetra/pentacyclic)
	**Other compounds** Monocyclic triterpene glycoside: Rhabdastoside A, Sterols, [71] Fatty acids, [72] gibepyrone F, gibepyrone C, *p*-hydroxy benzaldehyde, 3-Indole-3-aldehyde, thymine, *p*-hydroxy benzaldehyde, 3-indole-3-aldehyde *cyclo* (*S*-leucyl-*S*-prolyl), cyclo (L-Pro-L-Phe) (from bacterial symbiont [73]), cyclo (L-Pro-L-Leu) (from bacterial symbiont [73]), cyclo (D-Pro-D-Val) (from bacterial symbiont [74])
*Rhabdastrella* sp. ^3^ Taiwan	Rhabdastins H-I
*Rhabdastrella* sp. ^4^ Indonesia	Globostellatic acid X methyl esters, Globostellatic acid F methyl ester, 13-(*E*)-Globostellatic acid B methyl ester, Acetyljaspiferal E
*Geodia* sp. South China Sea	Geoditins A-B (23*E*) Stellettins A-B

^1^ Stellettins named after the sponge genus *Stelletta* are spelled with two ‘t’s [29]. However, they are regularly misspelled in the literature as ‘stelletins’. More confusing is that globostelletins were originally spelled with one ‘t’ [56]. ^2^ Stellettin N from Li et al. [61] is here renamed stellettin W, as stellettin N was already given to another compound by Xue et al. [58]. ^3^ This is probably *R. globostellata* according to the external morphology (Figure 3D), but we cannot be sure since there is no voucher. ^4^ This is potentially a new species of *Rhabdastrella* awaiting formal description.

## Data Availability

All species identifications generated during this study are available in Table 1 and Table S1.

## Supplementary Materials

The following supporting information can be downloaded at: https://www.mdpi.com/article/10.3390/md20030190/s1, Table S1: List of publications (1981–2022) reporting isomalabaricane triterpenes and derivatives from marine sponges.
