# Supplementary material for: Good Practices in Sponge Natural Product Studies: Revising Vouchers with Isomalabaricane Triterpenes
Source: Mar Drugs. 2022 Mar 4;20(3):190. doi: 10.3390/md20030190 (PMC8955210; doi:10.3390/md20030190)
Supplement: Supplementary file 1 [file marinedrugs-20-00190-s001.zip › marinedrugs-1626357-supplementary.pdf]

Table S1 List of publications (1981-2022) reporting isomalabaricane triterpenes and derivatives from marine sponges (sorted by date).

Original species identification. Lines in light green, vouchers originally identified as *Jaspis*. Lines in light blue, vouchers originally identified as *Stelletta*. Lines in light purple, vouchers originally identified as *Geodia*. Lines uncolored, vouchers originally identified as *Rhabdastrella*.

Compounds. In bold, new compounds reported. In black, isomalabaricane triterpene compounds; in red: sterols; in green: other types of compounds.

Abbreviations: CASIZ: California Academy of Sciences Invertebrate Zoology, San Francisco, CA, USA; CRRF: Coral Reef Research Foundation, Palau; GBR: Great Barrier Reef, E. Australia; IRD: Institut pour la Recherche et le Development, Nouméa, New Caledonia; PDZ, University of Utah collection, Salt Lake City, USA; QMG: Queensland Museum, Brisbane, Australia; UCMPW, University of California Museum of Paleontology, Berkeley, CA; USP, Regional Herbarium, School of Pure and Applied Sciences, University of the South Pacific, Fiji; WAM: Western Australian Museum; ZMAPOR: Amsterdam Porifera collection, now stored at Naturalis, Leiden, The Netherlands.

**Original species identification in ref.**	**Locality**	**Compounds**	**Sponge identifier and ref.**	**Description in ref.**	**Voucher**	**Revised Identification**	**Authorship of revised identification**
Jaspis stellifera	Suva, Fiji	**Stellettins ?, B, G** (B and G named retrospectively)	- Ravi, Wells & Croft (1981)	Ext: chocolate brown, Int: butter-color to intense yellow	-	*R. globostellata*	Kennedy (2000)
Jaspis stellifera	Heron Island, GBR	**4 triterpenes** including **stelliferin A-like**	- Ravi & Wells (1982)	-	-	*R. globostellata*	Kennedy (2000)
Stelletta sp.	Mogadishu, Somalia	Stellettin B	P. Bergquist, McCabe et al. (1982)	Ext: brown, Int: yellow	-	*R. globostellata*	This study (based on color)
Jaspis stellifera	Ishigaki Island, Okinawa, Japan	**Stelliferins A-F**	Jane Fromont, Tsuda et al. (1991)	Ext: brown, Int: bright yellow	-	*R. globostellata*	Kennedy (2000) and Jane Fromont, (pers. com).
Stelletta tenuis	Hainan, South China Sea	**Stellettin A** (yellow pigment)	Jin-He Li, Su et al. (1994)	-	#91-6 (not seen in this study)	*R. globostellata*	This study (based on identifier and authors)
Stelletta globostellata	Mage Shima Island, Japan	**Globostellatic acids A−D** **9-hydroxystelliferin D, 3-*epi*-29-hydroxystelliferin E, 3-*epi*-29-hydroxystelliferin A**	Rob van Soest, Ryu et al. (1996) Oku et al (2000)	-	ZMA POR 11016 (slide ZMAPOR P2724)	*R. globostellata*	This study (slide examined + deck picture)

					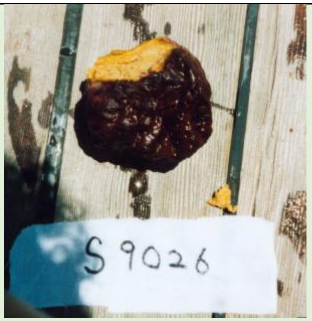<br><br>Picture courtesy from R. van Soest<br>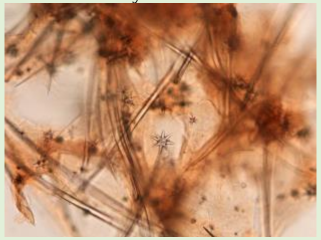<br>Picture from spicule slide		
Jaspis stellifera	Ishigaki Island, Okinawa, Japan	**Jaspiferals A-G***	Jane Fromont,<br>Kobayashi et al. (1996)	Ext: brown	-	*R. globostellata*	Kennedy (2000) and Jane Fromont, (pers. com).
Stelletta sp.	Cape Wilberforce, North Australia	**Stellettins C–F,**<br>stellettins A–B,<br>stellettin G	Shirley Pomponi<br>McCormick et al (1996),<br><br>McKee et al., (1997)	Spherical,<br>Ext: brown-purple, Int: brown yellow	Q66C4702 (Smithsonian)<br>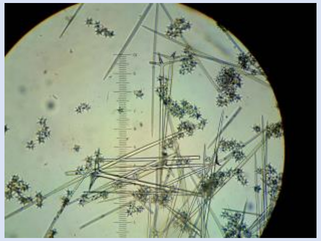<br>Picture from spicule slide, courtesy of C. Castello-Branco	*R. globostellata*	This study (based on description and pictures of the spicules sent by the Smithsonian NMNH)
R. globostellata	Hainan Island,<br>South China Sea	**Rhabdastrellic acid-A**<br>(= *E* isomer of stellettin G)	Rob van Soest,<br>Rao et al (1997)	yellow	SS301<br>= ZMAPOR 12451<br>(slide ZMAPOR P2703)	ID confirmed (‘brain’ morphotype)	This study (slide examined + deck picture)

					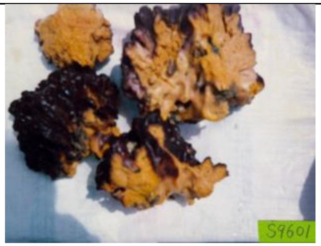<br><br>Picture courtesy from R. van Soest<br>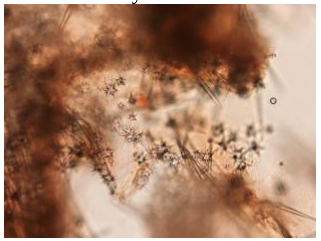<br>Picture from spicule slide		
R. globostellata	New Caledonia	**Aurorals 1-4**	Claude Lévi,<br>Bourguet-Kondracki et al. (2000)	-	IRD R1481<br>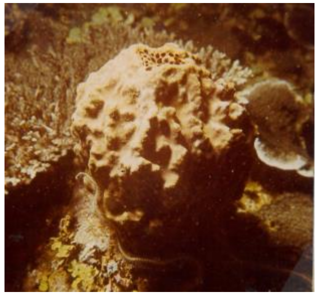<br>Picture courtesy from C. Debitus<br>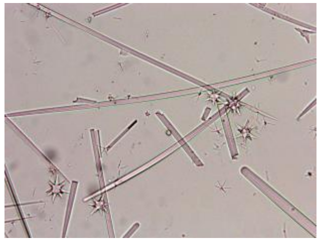	ID confirmed	This study (slide examined)

					Picture from spicule slide		
Jaspis sp.	Emae, Vanuatu Islands	**Globostellatic acid E,**<br>**3-*O*-acetyljaspiferal B–D and G,**<br>**jaspiferoic acids A–B dimethyl esters,**<br>globostellatic acids B–C	John N. A. Hooper,<br>Zampella et al. (2000)	-	QMG 306893 = IRD R1624<br>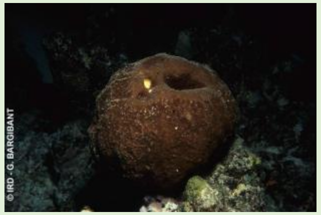<br><br>Pictures of spicules: cf. Kennedy, 2000, Fig. 7)	*R. globostellata*	Kennedy (2000) and this study (pictures of spicules)
Geodia globostellifera	Vanua Levu, Fiji	**Stelliferin A riboside** (=3-*Epi*-stelliferin A 22-α-ribopyranoside)**,**<br>stellettins A-B	John N. A. Hooper,<br>Tabudravu & Jaspars (2001)	-	USP 9712SD114<br>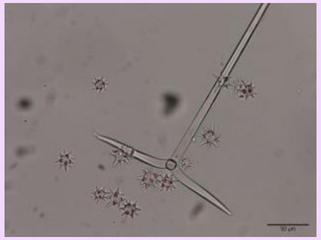<br>Picture from spicule slide	*R. globostellata*	Mary Kay Harper in Tasdemir et al. (2002) and Cárdenas et al. (2011), specimen examined and barcoded (COI).
Geodia japonica	Xisha Island,<br>Hainan,<br>South China Sea	**geoditins A–B*,**<br>stellettins A–B,<br>**26- methylergosta-5,24(28)-dien-7-one-3-ol,**<br>24(28)dehydroaplysterol, 24-methylenecholesterol,<br>*cyclo*(*S*-leucyl-*S*-prolyl)**,**<br>palmitic acid,<br>malonic acid	Li Chupu,<br>Zhang & Che (2001)<br><br>+ see activity of Stellettin A (Liu et al., 2005; 2006; 2012)	-	ZWH95004<br>(lost, Prof. Jun Xu, pers. comm.)	*Geodia* sp.	This study<br>(*G. japonica* is a temperate, cold water species so this is probably a mis-identification).
Jaspis sp.	Vava'u, Tonga,<br>South Pacific	**29-hydroxystelliferin E,**<br>**29-hydroxystelliferin A,**<br>**stelliferin G,**<br>3-*epi*-29-hydroxystelliferin E	Michelle Kelly,<br>Meragelman et al. (2001)	-	CRRF 0CDN5445-C<br>= CASIZ 300204	*R. globostellata*	Michelle Kelly in Tasdemir et al. (2002)

					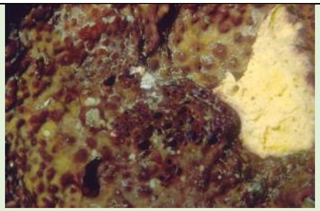<br>Picture courtesy of CRRF		
R. globostellata	Mindanao, Philippines	**Stellettins H–I**,<br>stellettins A–D,<br>(–)-stelettin E,<br>rhabdastrellic acid-A	Mary Kay Harper,<br>Tasdemir et al (2002)	-	PDZ_1 98-1-10<br>(University of Utah)<br>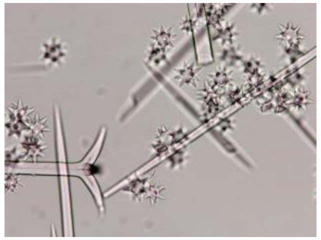<br>Picture from spicule slide	*Rhabdastrella* cf. *globostellata*	Cárdenas et al. (2011), specimen examined and barcoded (COI).
R. aff. *distincta*	Hainan Island,<br>South China Sea	**Isogeoditin A,**<br>**13-(*E*)-isogeoditin A,**<br>**isogeoditin B,**<br>**22,23-dihydrostellettin B**,<br>geoditins A–B,<br>stellettins A–C, E,<br>rhabdastrellic acid-A<br><br>**Rhabdastrellins A−F,**<br>stellettins L–M	Rob van Soest,<br>Lv et al. (2004)<br><br>Lv et al. (2008)	-	HS-14 (Peking University)<br>= ZMAPOR 2717<br>(slide ZMAPOR P16728)<br>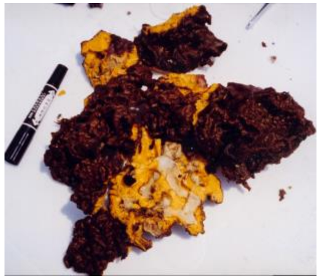<br>Picture courtesy from R. van Soest	*R. globostellata*	This study (slide examined + deck photo)

					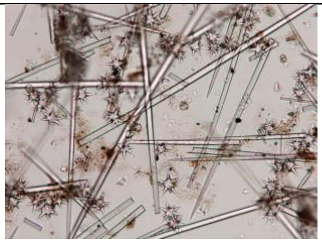<br>Picture from spicule slide		
Jaspis sp.	Sanya,<br>Hainan,<br>South China Sea	**22,23-dihydrostellettin D**<br><br>**Jaspolides A–F**<br><br>**Jaspolides G–H**<br>(bisisomalabaricane)	Rob van Soest?<br>Tang et al. (2005)<br><br>Tang et al. (2006)<br><br>Tang et al. (2007)	-	HSC-39<br>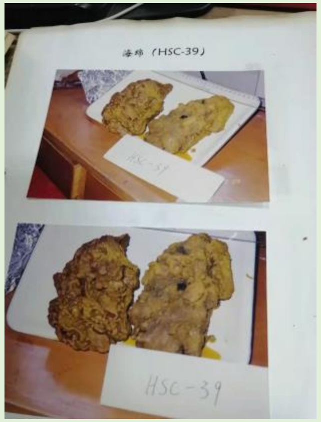<br><br>Picture courtesy of Wenhanlin.<br>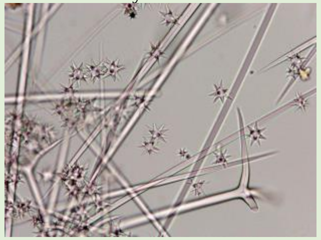<br>Picture from spicule slide	*R. globostellata*	This study<br>(slide examined)
R. globostellata	Fiji	**Stellettins J–K**,<br>stelliferin A riboside,<br>3-epi-29-acetoxystelliferin E	Michelle Kelly,<br>Clement et al. (2006)	picture in Supp. Mat.	CRRF 0CDN4278-K (deposited at NIWA)	ID confirmed<br>(pink morphotype)	This study<br>(picture + voucher examined by M. Kelly)

					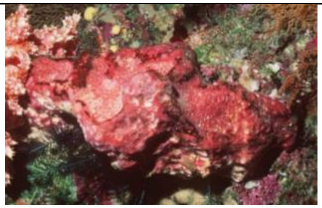<br>Picture from CRRF		
R. globostellata	South Sulawesi, Indonesia	**Globostelletin, globostellatic F–M,**<br>**two new stelliferin A ribosides,**<br>globostellatic acids A, D,<br>stelliferin A riboside	Rob van Soest,<br>Fouad et al (2006)	SpherastersI (20-55)<br>Spherasters II (10-15)<br>Oxyasters/tylasters<br>Trichodragmas (100x5-12)	ZMAPOR17166<br>(slide ZMAPOR P2693)<br>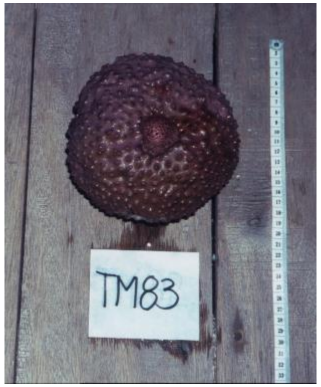<br><br>Picture courtesy from R. van Soest.<br>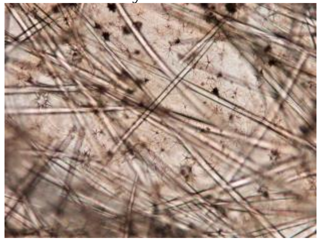<br>Picture from spicule slide	ID confirmed	This study (slide examined + deck photo)
R. globostellata	Pith Reef, GBR	**Stelliferin D riboside**,<br>stelliferin A,<br>stelliferin A-like	-<br>Agrawal (2007), PhD thesis	-	-	-	-

R. globostellata	Sulawesi, Indonesia	**Globostellatic acids X methyl ester 1-4,<br>Globostellatic acid F methyl ester,<br>13*E*-Globostellatic acid B methyl ester,<br>Acetyljaspiferal E**	Nicole de Voogd,<br>Aoki et al (2007)	-	ZMAPOR 15784a<br>(slide ZMAPOR P2697)<br>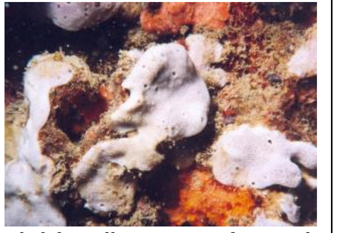<br>*Rhabdastrella* covered with *Haliclona (Gellius) amboinensis* (Picture courtesy from N. de Voogd)<br>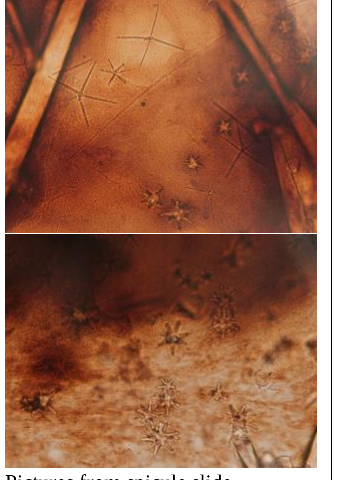<br>Pictures from spicule slide	*Rhabdastrella* sp.	This study (slide examined+ underwater picture)
Stelletta tenuis	Sanya Bay,<br>Hainan,<br>South China Sea	**Stellettins L–M,**<br>stellettins A–E,<br>stellettin H,<br>rhabdastrellic acid-A,<br>22,23-dihydrostellettin B,<br>geoditin A<br>**24-methylene-27-methylcholestane-3β,5α,6β-triol,**<br>**24-methylene-27-**	Jin-He Li,<br>Lin et al. (2007)	-	HN1120-1<br>(not seen in this study)	*R. globostelletta*	This study (according to another voucher examined, identified by Jin-He Li as *S. tenuis*)

		**methylcholest-5-ene-3β,7α-diol,**<br>**24-methylene-27-methylcholest-5-ene-3β,7β -diol,**<br>24-methylene-27-methylcholest-5-en-3β-ol-7-one,<br>24-methylene-27-methylcholesterol,<br>24,25-dimethylcholestane-2β,3α,3α-triyl trisodium sulfate					
R. globostellata	Amami-oshima, Japan	**Rhabdastins A−G,**<br>**Rhabdastoside A**<br>**(**triterpene glycoside)	Rob van Soest,<br>Hirashima et al (2010)	-	ZMAPOR 16401<br>(slide ZMAPOR P2690)<br>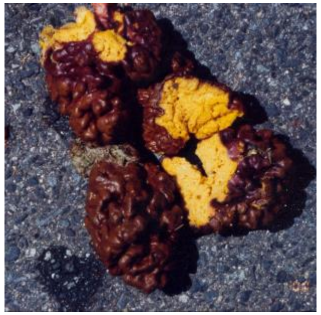<br>Picture courtesy from R. van Soest<br>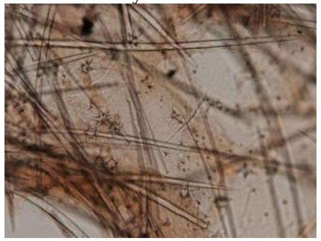<br>Picture from the spicule slide	I.D. confirmed<br>('brain' morphotype)	This study<br>(slide examined + deck picture)
R. globostellata	Hainan Island,<br>South China Sea	**Globostelletins A–I,**<br>jaspolide F,<br>(–)-stellettin E,<br>stellettins C–D rhabdastrellic acid-A<br><br>**Globostelletins J–S**	Nicole de Voogd,<br>Li et al (2010)<br><br><br><br>Li et al (2012)	Low res. picture of HSF-10 in article banner	HSF-10<br><br>= ZMAPOR 17354<br>(slide ZMAPOR P2700)	ID confirmed	This study<br>(slide examined)

					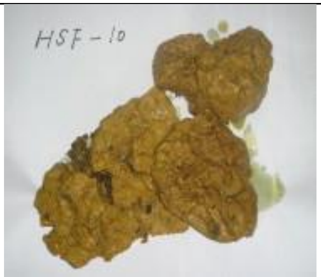<br>Picture courtesy from Li et al.<br>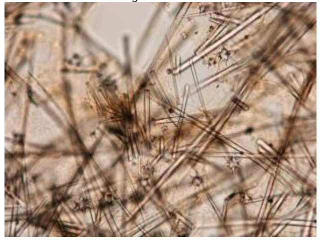<br>Picture from spicule slide		
R. cf. *globostellata*	Ishigaki Island, Okinawa, Japan	**Stelliferins J–N**	Jane Fromont,<br>Tanaka et al (2011)	Yellowish-brown, description of spicules	WAM Z59146<br>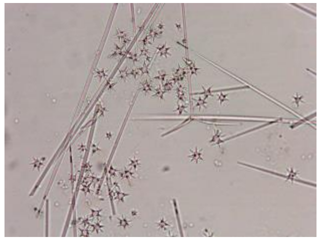<br>Picture from spicule slide	*R. globostellata*	This study (voucher examined + COI)
Jaspis stellifera	Guangdong,<br>South China Sea	**Jaspiferins A–B**<br>gibepyrone F,<br>*p*-hydroxy benzaldehyde,<br>3-Indole-3-aldehyde,<br>thymine,<br>24(28)-dehydroaplysterol,<br>(25*S*)-26-methylene-cholest-4-en-3-one	-<br>Tang et al (2012)	-	XWS-03<br>(not seen in this study)	*Rhabdastrella* sp.	This study (*J. stellifera* is not found in South China Sea, the compounds indicate it is probably a *Rhabdastrella*)
Stelletta sp.	Lingshui Bay, Hainan Province, China	**Stellettin N,**<br>stellettins D, G, H,	Jin-He Li,<br>Xue et al (2013)	-	YAL-37	*R. globostelletta*	This study (picture)

		Rhabdastrellic acid-A,<br>22,23-dihydrostellettin D			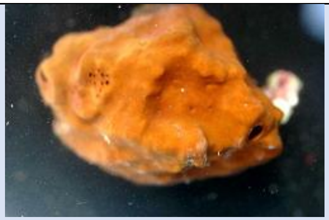<br>Picture courtesy of Xue et al.		
Jaspis stellifera	South China Sea	**Jaspiferins C–F**<br><br>**Jaspiferin G**<br>isogeoditin A,<br>13-(*E*)-isogeoditin A, jaspolide E, 22,23-dihydrostellettin D<br><br>**Jaspiferin H-J,**<br>stellettin E, F	Sheng-An Tang,<br>Jin et al (2014)<br><br>Xu et al (2016)<br><br>Xu et al (2018)	-	SC20090608<br>(not seen in this study)	*Rhabdastrella* sp.	This study (*J. stellifera* is not found in South China Sea, the compounds indicate it is probably a *Rhabdastrella*)
Stelletta tenuis	Sanya Bay,<br>Hainan,<br>South China Sea	**Stellettins O–P, W[1],**<br>stellettins C–D,<br>gibepyrone C,<br>gibepyrone F<br><br>[1]stellettin N was already given to another stellettin by Xue et al. (2013). This one is here renamed stellettin W.	Jin-He Li,<br>Li et al (2015)	-	HN1120-5<br>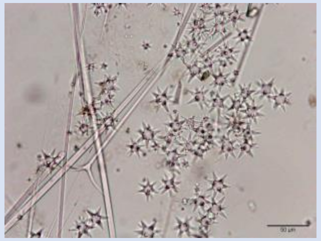<br>Picture from spicule slide	*R. globostelletta*	This study<br>(voucher examined)
Rhabdastrella providentiae	Con Co, Quang Tri,<br>Vietnam	**Rhabdastrellins G-K**<br>jaspolide C,<br>globostelletin C,<br>globostelletin D<br><br>**Rhabdaprovidines A-C**<br>**(**formed by bio-chemical degradation of ITTs?)<br><br>**Rhabdaprovidines D-G**	Do Cong Thun,<br>Kiem et al (2018)<br><br>Dung et al. (2018a)<br><br>Dung et al. (2018b)	picture	HM06.2016-01<br>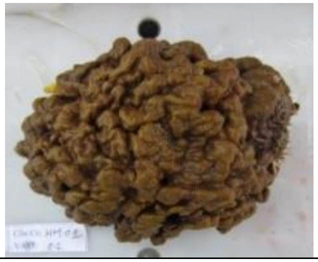	*R. globostellata*<br>(‘brain’ morphotype)	This study<br>(voucher examined)

					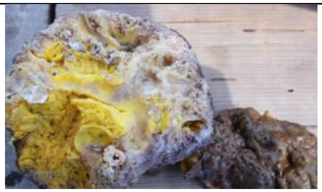<br>Picture courtesy of Kiem et al.<br>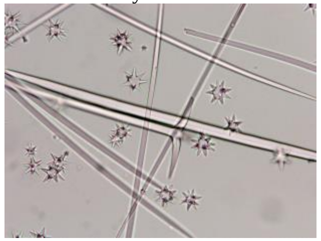<br>Picture from spicule slide		
Stelletta sp.	Cham Island, Vietnam	**Cyclobutastellettolides A-B**,<br>jaspolide F,<br>globostelletins E-G, K, M<br><br>**Stellettins Q-R**,<br>**stellettins S-V,**<br>globostelletin N	Boris B. Grebnev,<br>Kolesnikova et al. (2019)<br><br>Kolesnikova et al. (2021)	picture and complete description of spicules	PIBOC O38-301<br>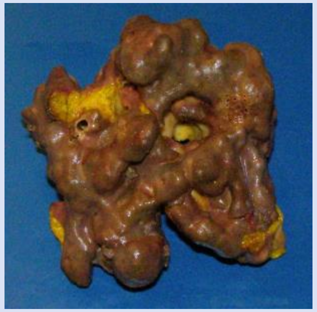<br>Picture courtesy of Kolesnikova et al.<br>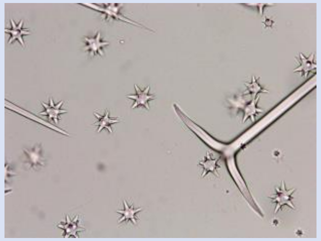<br>Picture from spicule slide	*R. globostelletta* (‘brain’ morphotype)	This study (voucher examined)

Rhabdatrella sp.	Kenting, Taiwan	**Rhabdastin H-I**	Hsing-Hui Li,<br>Lai et al. (2021)	picture (not the one published), 18S (lost)	#2017-1221-SP<br>(but wrong voucher)<br>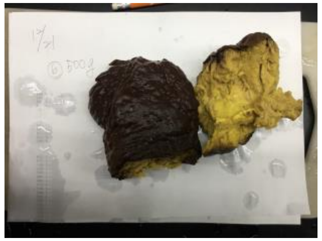<br>Picture courtesy of Lai et al.	*Rhabdastrella* sp.	This study (picture)
R. globostellata	Vanphong Bay, Khanh Hoa, Vietnam	**Rhabdaglostelones A-C**	-<br>Trang et al. (2022)	COI and 18S markers (in Suppl. Info.)	NCCT-B139<br>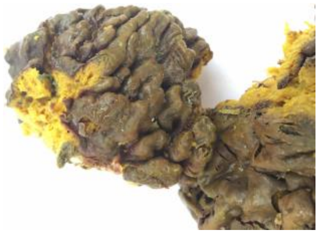<br>Picture courtesy of P. V. Kiem<br>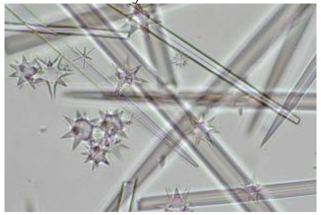<br>Picture from spicule slide	I.D. confirmed ('brain' morphotype)	This study (voucher examined)
